# Exploring complex cellular phenotypes and model-guided strain design with a novel genome-scale metabolic model of *Clostridium thermocellum* DSM 1313 implementing an adjustable cellulosome

**DOI:** 10.1186/s13068-016-0607-x

**Published:** 2016-09-06

**Authors:** R. Adam Thompson, Sanjeev Dahal, Sergio Garcia, Intawat Nookaew, Cong T. Trinh

**Affiliations:** 1 Bredesen Center for Interdisciplinary Research and Graduate Education, University of Tennessee, Knoxville, TN 37996 USA; 2 Oak Ridge National Laboratory, Oak Ridge, TN 37831 USA; 3 BioEnergy Science Center, Oak Ridge National Laboratory, Oak Ridge, TN 37831 USA; 4 Comparative Genomics Group, Biosciences Division, Oak Ridge National Laboratory, Oak Ridge, TN 37831 USA; 5 Department of Chemical and Biomolecular Engineering, University of Tennessee, 1512 Middle Dr., DO#432, Knoxville, TN 37996 USA; 6 Department of Biomedical Informatics, College of Medicine, University of Arkansas for Medical Sciences, Little Rock, AR 72205 USA

**Keywords:** *Clostridium thermocellum*, Genome-scale model, Consolidated bioprocessing, Biofuels, Bioenergetics, Elementary mode analysis, Flux balance analysis, Rational strain design, Minimal cut sets

## Abstract

**Background:**

*Clostridium thermocellum* is a gram-positive thermophile that can directly convert lignocellulosic material into biofuels. The metabolism of *C. thermocellum* contains many branches and redundancies which limit biofuel production, and typical genetic techniques are time-consuming. Further, the genome sequence of a genetically tractable strain *C. thermocellum* DSM 1313 has been recently sequenced and annotated. Therefore, developing a comprehensive, predictive, genome-scale metabolic model of DSM 1313 is desired for elucidating its complex phenotypes and facilitating model-guided metabolic engineering.

**Results:**

We constructed a genome-scale metabolic model *i*AT601 for DSM 1313 using the KEGG database as a scaffold and an extensive literature review and bioinformatic analysis for model refinement. Next, we used several sets of experimental data to train the model, e.g., estimation of the ATP requirement for growth-associated maintenance (13.5 mmol ATP/g DCW/h) and cellulosome synthesis (57 mmol ATP/g cellulosome/h). Using our tuned model, we investigated the effect of cellodextrin lengths on cell yields, and could predict in silico experimentally observed differences in cell yield based on which cellodextrin species is assimilated. We further employed our tuned model to analyze the experimentally observed differences in fermentation profiles (i.e., the ethanol to acetate ratio) between cellobiose- and cellulose-grown cultures and infer regulatory mechanisms to explain the phenotypic differences. Finally, we used the model to design over 250 genetic modification strategies with the potential to optimize ethanol production, 6155 for hydrogen production, and 28 for isobutanol production.

**Conclusions:**

Our developed genome-scale model *i*AT601 is capable of accurately predicting complex cellular phenotypes under a variety of conditions and serves as a high-quality platform for model-guided strain design and metabolic engineering to produce industrial biofuels and chemicals of interest.

**Electronic supplementary material:**

The online version of this article (doi:10.1186/s13068-016-0607-x) contains supplementary material, which is available to authorized users.

## Background

For a sustainable energy economy, the necessity of producing fuels and chemicals from renewable feedstocks is well acknowledged, and the use of bio-based resources is a promising route for significantly lowering the carbon footprint of liquid transportation fuels [1]. To produce economically favorable biofuels and chemicals, consolidated bioprocessing (CBP) is advantageous as it utilizes specialized micro-organisms for direct conversion of lignocellulosic biomass into target chemicals in a single step [2–4].

Of particular interest for CBP is the gram-positive thermophile *Clostridium thermocellum*, which exhibits a high growth rate on cellulose [5, 6] and can endogenously produce the biofuels ethanol [7], hydrogen [8], and isobutanol [9]. These desirable phenotypes are feasible because *C. thermocellum* possesses a large, organized, extracellular cellulosome [10, 11] which is highly efficient at degrading lignocellulosic materials [12]. *Clostridium thermocellum* also contains an intricate, robust system of branched catabolic pathways that recycle reduced ferredoxin and NAD(P)H for cell growth and lignocellulose degradation [13]. This branched metabolism, however, makes production of a single product such as ethanol in *C. thermocellum* quite challenging.

Recently, there has been extensive work towards engineering *C. thermocellum* for increased ethanol production, e.g., (i) elimination of acetate production [14], (ii) elimination of lactate production [15], (iii) elimination of both acetate and lactate production [16], (iv) elimination of hydrogen production [17], (v) elimination of formate production [18], (vi) elimination of all the aforementioned traditional fermentation products [19], and (vii) elimination of malic enzyme activity while expressing an endogenous pyruvate kinase [20]. Despite these efforts, ethanol yield is still below industrially relevant levels. In the best performing strain, a yield above 70 % theoretical maximum has only been demonstrated at low substrate loadings [19], and ethanol yield dropped when substrate concentrations were increased [9, 21]. These reports open many questions into the robustness of *C. thermocellum* redox metabolism and how regulatory mechanisms lead to the observed phenotypes in both cellobiose- and cellulose-grown cultures.

Constraint-based genome-scale metabolic modeling is rapidly becoming a standard tool for investigating cellular metabolism. The information contained in a genome sequence is redefined as a series of mass- and charge-balanced reactions in a genome-scale metabolic model (GEM). When coupled with thermodynamic constraints, metabolic flux constraints (e.g., substrate uptake rates and/or product secretion rates), and a cellular objective, GEM analysis can determine metabolic flux distributions, i.e., cellular phenotypes, under specified growth conditions. A repertoire of metabolic pathway analysis tools based on flux balance analysis and elementary mode analysis has recently been developed to analyze these GEMs and have been extensively reviewed [22–25]. A *C. thermocellum* GEM *i*SR432 has been constructed previously [26], used as a scaffold for transcriptomic constraints [27], and structurally compared to a number of other *Clostridial* GEMs [28]. While useful, recent results highlight several limitations of *i*SR432, e.g., (i) there have been many advancements in the knowledge of *C. thermocellum* atypical glycolysis [29], pentose phosphate pathway [30], and redox metabolism redundancies [31, 32] which were not included in the original model, (ii) the model was constructed for the strain ATCC 27405, but not DSM 1313 [33], which is the genetically tractable parent strain used in metabolic engineering strategies [34], (iii) the model included a cellulosome term but it was not variable with respect to carbon source, which has been shown to vary substantially [35], and (iv) the model did not accurately predict certain cellular phenotypes like ethanol production [26].

In this work, we constructed a new GEM for DSM 1313 from the KEGG database, expanding upon our previously constructed central metabolic model [36], and manually curated the GEM with the most current knowledge of *C. thermocellum* metabolism. We next refined the GEM using several sets of high-quality batch fermentation data for cell growth on various carbon sources, i.e., cellobiose and cellulose. This is accomplished by first tuning the energetic requirements for growth on cellobiose, then finding the additional ATP cost of producing the cellulosome for growth on cellulose. With this validated model, we investigated a series of interesting observations presented in literature. First, we reproduced the difference in cell yields with respect to cellodextrin lengths, a direct consequence of the phosphorolytic sugar assimilation mechanism of *C. thermocellum* [37, 38]. Next, we used the model to predict metabolic engineering strategies to enhance the production of the desirable biofuels ethanol, hydrogen, and isobutanol for future experimental study.

We concluded the story using the model to investigate how *C. thermocellum* metabolism changes when growing on cellobiose versus cellulose. Using literature reports and flux sampling, we elucidated a regulatory mechanism to explain why cultures growing on cellulose do not reach the ethanol yields of cultures growing on cellobiose, and illustrated how the robust energy and redox metabolism of *C. thermocellum* dramatically adapt to environmental growth perturbations.

## Results

### Model construction and comparison

Following the construction process outlined in “Methods”, we obtained the *C. thermocellum* DSM 1313 GEM, named *i*AT601 following convention [26, 39]. This new model presents a significant improvement from the existing ATCC 27405 GEM *i*SR432 [26] by incorporating very recently expanded knowledge of *C. thermocellum* metabolism. In particular, we updated the cofactor specificity of glycolytic enzymes [29] based on in vitro protein characterization as well as performed Cofactory analysis [40] to resolve cofactor specificity when in vitro data was unavailable (see “Methods” section). We also manually curated the intricate carbon overflow and redox metabolisms with recently acquired knowledge [9, 36].

Importantly, we built the GEM *i*AT601 to account for the composition and synthesis cost of the cellulosome because *C. thermocellum* is known to alter cellulase expression when cultured on different carbon sources (e.g., cellobiose, cellulose, switchgrass, etc.) and/or at different growth rates [35, 41, 42]. To construct the cellulosome term for the GEM *i*AT601, we compiled experimentally measured protein and amino acid distributions for the cellulosome during growth on different cellulosic substrates [43]. While protein compositions of the cellulosomes (e.g., hydrolyases, scaffodins, dockerins, etc.) significantly changed for growth on different substrates, amino acid compositions of these cellulosomes remained relatively similar (Additional file 2: Figure S1). FBA simulations using amino acid compositions of various cellulosomes and a maximum growth objective gave similar values of predicted optimal growth within 0.2 % variation. Thus, we used the median amino acid requirement across the different culture conditions for the cellulosome term in the GEM *i*AT601.

**Fig. 1:**
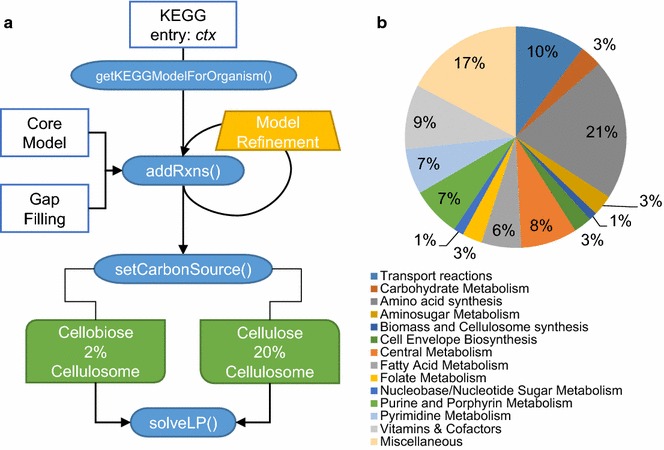
**a** Flowchart of model construction. **b** Distribution of *i*AT601 reactions belonging to KEGG pathways

Overall, the GEM *i*AT601 contains 872 reactions, 904 metabolites, and 601 genes. Included in the model are 114 transport and exchange reactions for the 57 extracellular metabolites (Table 1). This represents an increase in reactions, metabolites, and genes by 51, 72 and 39 %, respectively, over *i*SR432 [26]. The GEM *i*AT601 encompasses all major metabolic pathways of *C. thermocellum*, and the numbers of reactions within different KEGG pathways are summarized in Fig. 1.

**Table 1:** Comparison of the GEM attributes among various *Clostridial* species

Properties	*Clostridial* genome-scale metabolic models				
	*i*CAC490	*i*CM926	*i*FS431	*i*SR432	*i*AT601
	*C. acetobutylicum*	*C. beijerinckii*	*C. cellulolyticum*	*C. thermocellum*	*C. thermocellum*
	DSM 824	NCIMB 8052	H10	ATCC 27405	DSM 1313
ORFs	4017	5100	3575	3238	3033
Reactions	794	938	621	577	871
Transport	120	68	45	73	110
Included genes	490	925	431	432	600
Metabolites	707	821	603	525	904
Reference	[97]	[76]	[77]	[26]	This study

### ATP requirement for growth on cellobiose

**Fig. 2:**
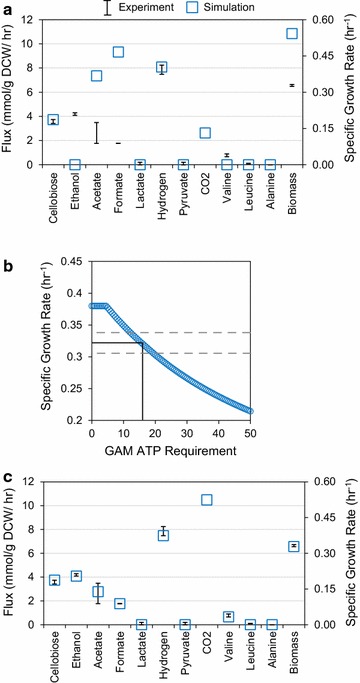
**a** Comparison of experimental and simulated metabolic fluxes for optimal growth of *C. thermocellum* on cellobiose without tuned growth-associated maintenance (GAM) ATP requirement. FBA simulation used only experimentally determined cellobiose uptake as a constraint. **b** Identification of best-fit GAM ATP requirement. The model energy balance was tuned by altering GAM ATP requirement and optimizing growth rate with specified fermentation constraints. *Dotted lines* frame the experimentally observed growth rate range, while *solid lines* illustrate the average observed growth rate and the best-fit GAM ATP requirement. **c** Comparison of experimental and simulated metabolic fluxes for optimal growth of *C. thermocellum* on cellobiose with tuned GAM ATP requirement

After construction, we proceeded to train the model using pH-controlled batch fermentation data collected for the wild-type DSM1313 grown on MTC defined media with cellobiose as a carbon source [36, 44]. Table 2 presents the experimental fluxes used to constrain the model, and for all simulations, a non-growth-associated maintenance (NGAM) cost of 3.27 mmol ATP/g DCW/h was used [38]. We first investigated the model’s growth predictions with the cellodextrin uptake rate as a sole flux constraint. For cellobiose-grown simulations, the model did not predict any ethanol production under maximum growth conditions. Figure 2a shows the predicted phenotype for all major fermentation products under this initial condition. Immediately noticeable is that maximizing cell growth correlated with an overestimation of acetate production, presumably due to the additional ATP produced by the phosphotransacetylase–acetate kinase pathway. The acetate overestimation was also associated with high formate production to balance the redox state of the cell in silico, but these simulated results were clearly not consistent with the in vivo phenotype. Since the model predicted faster growth than observed experimentally and our initial biomass composition only contained the ATP requirements for biopolymer synthesis [45], it is clear that the growth-associated maintenance (GAM) cost must be refined.

**Table 2:** Experimental fluxes used for metabolic model constraints

Experimental flux (mmol species/g DCW/h)	Cellobiose	Cellulose
r_CB up_	3.58 ± 0.16	–
r_GluEq_up_	–	6.39 ± 0.08
r_ETH_	4.19 ± 0.10	2.63 ± 0.03
r_ACE_	2.63 ± 0.86	3.40 ± 0.15
r_FOR_	1.77 ± 0.01	1.38 ± 0.01
r_H2_	7.86 ± 0.38	nd
r_VAL_	0.78 ± 0.12	nd
r_LAC_	0.18 ± 0.01	0.00 ± 0.0
μ (1/h)	0.33 ± 0.01	0.31 ± 0.01
Reference	[36]	[44]

*nd* not determined

While it is straightforward to calculate the ATP required to synthesize 1 g of dry cell weight [45, 46], the extra requirement for GAM (i.e., for regulation of cellular osmotic level, protein secretion, and flagellar motion) is less straightforward and is normally calculated with substrate-limited chemostat experiments [47]. Since the GAM is typically condition dependent [48] and industrially relevant conditions are not carbon-limited [49], we used the model to estimate an appropriate GAM coefficient.

To find the GAM, we set experimentally measured fluxes as constraints (Table 2) and varied an ATP requirement in addition to DCW synthesis while optimizing cell growth. A value of 13.5 mmol ATP/g DCW/h was found to best fit growth on cellobiose in batch conditions (Fig. 2b). When maximizing growth under the experimentally obtained flux constraints together with the tuned GAM coefficient, the model matched well with the experimental growth and fermentation profile (Fig. 2c). Further, with this GAM parameter the model was used to predict the growth of several mutants while constraining fermentation product fluxes to previously reported values [36], and the model predictions correlated very well with the experimentally observed growth rates (see Table S1 of Additional file 2).

### Additional ATP requirement for cellulosome synthesis

**Fig. 3:**
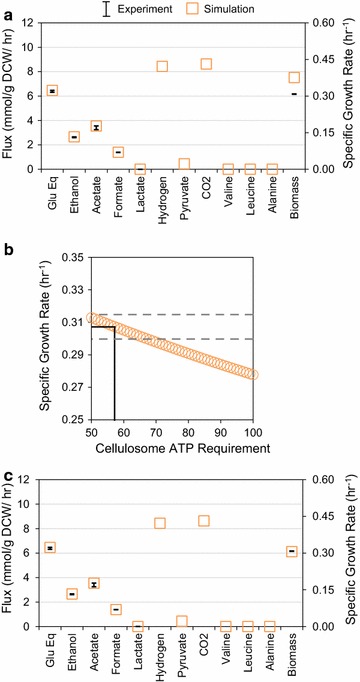
**a** Comparison of experimental and simulated metabolic fluxes for optimal growth of *C. thermocellum* on cellulose without ATP requirement for cellulosome synthesis. FBA simulation used experimentally measured fermentation product fluxes and the calculated GAM ATP requirement as constraints. **b** Identification of best-fit ATP requirement for cellulosome biosynthesis. The cellulosome ATP requirement was varied and growth rate was optimized with specified fermentation constraints. *Dotted lines* frame the experimentally observed growth rate range, while *solid lines* illustrate the average observed growth rate and the best-fit cellulosome ATP requirement. **c** Comparison of experimental and simulated metabolic fluxes for optimal growth of *C. thermocellum* on cellulose. FBA simulation used all experimental flux values as well as the best fit for GAM and cellulosome synthesis ATP requirements as constraints

We next performed in silico analysis of *C. thermocellum* growth on cellulose. It has been shown experimentally that the cellulosome is no longer suppressed as when *C. thermocellum* grows on cellobiose [35], and so we increased the percent of dry cell weight attributed to the cellulosome and applied experimentally measured flux constraints for simulation (Table 2). Using the previously calculated GAM value for growth on cellulose, however, still returned an over-estimated prediction of cell growth when specific fermentation rates were included as constraints (Fig. 3a). Since the cellulosome is a large, extracellular enzyme complex, the discrepancy between the model’s prediction and experimental observation was likely due to not accounting for an increased ATP demand for cellulosome synthesis and secretion.

To further train the GEM *i*AT601, we set the GAM and NGAM as described above while similarly increasing the ATP requirement for cellulosome synthesis and secretion to simulate maximum growth rates. We found that an ATP cost of 57 mmol ATP/g cellulosome/h was the best fit to wild-type growth on cellulose (Fig. 3b). This corresponds to 14 mmol ATP/g cellulosome/h greater than what is required for the cell protein synthesis (Additional file 1). Given that the cellulosome represents a greater proportion of the dry cell weight for growth on cellulose than cellobiose [35], this ATP cost is an equivalent overall increase of 1.14 and 11.4 mmol ATP/g DCW/h for cellobiose and cellulose simulations, respectively. By applying the ATP cost and fermentation rates as constraints, simulations of cell growth on cellulose matched very well with experimental data (Fig. 3c). Reapplying the cellulosome ATP cost to the previous cellobiose simulations did not alter the results outside of the experimentally observed flux ranges, and so for all further studies the GAM and cellulosome ATP coefficients are fixed at these values.

### Application of GEM for rational strain design

One important application of the tuned GEM *i*AT601 is to guide strain engineering for enhanced production of chemicals of interest. For instance, the constrained minimal cut set (cMCS) method [50] can be used to identify all feasible genotype variants with minimum metabolic functionalities tailored for production of specific chemicals [51]. While ethanol is a valuable product, there is also interest in engineering *C. thermocellum* to produce isobutanol [9, 52] or hydrogen [8, 53]. Using the cMCS method for genome-scale models [54], we investigated the feasibility of strain design for the production of ethanol, hydrogen, and isobutanol (Table 3; Additional file 3).

**Table 3:** Overview of strain designs from the minimal cut set algorithm

Products	Target cut set sizes	# Strain designs
Ethanol	6	67
	7	185
Hydrogen	4	12
	5	221
	6	1105
	7	4816
Isobutanol	7	28

Based on the tuned GEM *i*AT601, we found 67 unique cut sets of size 6 and 185 cut sets of size 7 that could produce high ethanol yields (i.e., at least 60 % of the maximum theoretical yield, see “Methods”) while tightly coupling with cell growth. As anticipated, many of the highly represented reactions are associated with the central metabolism, in particular redox metabolism (see Supplementary Figure S2 of Additional file 2), where redundancies in the network are eliminated to redirect carbon. For example, a common knockout strategy is the removal of 3-phosphoglycerate phosphomutase, thereby generating 2-phosphoglycerate (2PG) by hydrolysis of 3-phosphoglycerate to glycerate, then synthesizing 2PG by hydrolyzing ATP. This reaction cycle would slow growth by increasing ATP costs, and push more carbon towards ethanol. Another interesting strategy which arose from the cMCS calculations is the removal of urea metabolism, which could lead the way for media optimization strategies in the future. These results are indicative of the level of redundancy within *C. thermocellum* metabolism, and provide perspective on shortcomings of previously reported metabolic engineering strategies.

For hydrogen production, we found many solutions, including 12 intervention strategies of size 4 and 4816 strategies of size 7. The presence of strategies of size 4 implies that fewer modifications are needed for high hydrogen production compared to ethanol, which requires a minimum of six modifications. Finally, for isobutanol we only found 28 strategies of size 7, hinting that high isobutanol production in *C. thermocellum* will be a challenge due to greater modifications required. Many of these metabolic engineering strategies are not trivial, and though the objective of this study is not to go into depth for these strategies, they are expected to be useful in guiding experimental implementations in future studies.

### Effect of cellodextrin lengths on growth

We next employed the GEM *i*AT601 to validate interesting cellular phenotypes of *C. thermocellum*. It has been shown experimentally that *C. thermocellum* prefers longer cellodextrins with an average glucose length of ~4.2 and can assimilate up to cellohexaose (G6). In addition, the cell yield was observed to increase with longer cellodextrins supplied as a carbon source [38, 55, 56].

**Fig. 4:**
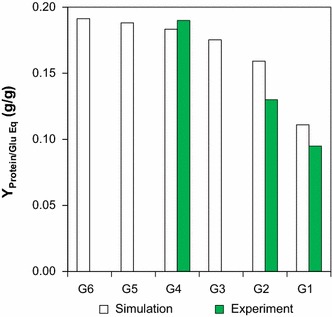
Comparison of effect of cellodextrin lengths on yield of cell protein per glucose equivalent (g/g) during simulation with *i*AT601 and values reported in the literature [55]. For culture simulations, a fixed glucose equivalent uptake rate of 6.5 mmol/g DCW/h was used. Cellodextrins of length N are shown as GN

To investigate the effect of assimilating various (G2–G6) cellodextrins and glucose (G1) on cell yields, we set the glucose-equivalents uptake flux at a constant 6.5 mmol/g DCW/h while altering the sole carbohydrate species available. To allow for direct comparison with experimental results [55], the simulation results are presented as yield of protein per glucose (g/g), where protein yield was calculated as the sum of fluxes to cellulosome and cell protein production (g proteinaceous component/g DCW/h) divided by the glucose-equivalents uptake flux (g glucose equivalents/g DCW/h). Our simulation shows that the maximum protein yield obtainable with G4 was 95 % of that obtainable on G6 while yields on G3, G2 and G1 dropped to around 92, 83 and 58 %, respectively, of the maximum under experimental conditions tested (Fig. 4). This drop in maximum protein yields with respect to shorter cellodextrins matched well with the experimental data (Fig. 4) [55]. This trend clearly follows the calculated bioenergetic benefit to assimilation of longer cellodextrins [38], and the result establishes confidence in the model’s bioenergetic constraints related to sugar assimilation.

### Effect of cellulosic substrates and cell growth rates on bioenergetics of *C. thermocellum*

**Fig. 5:**
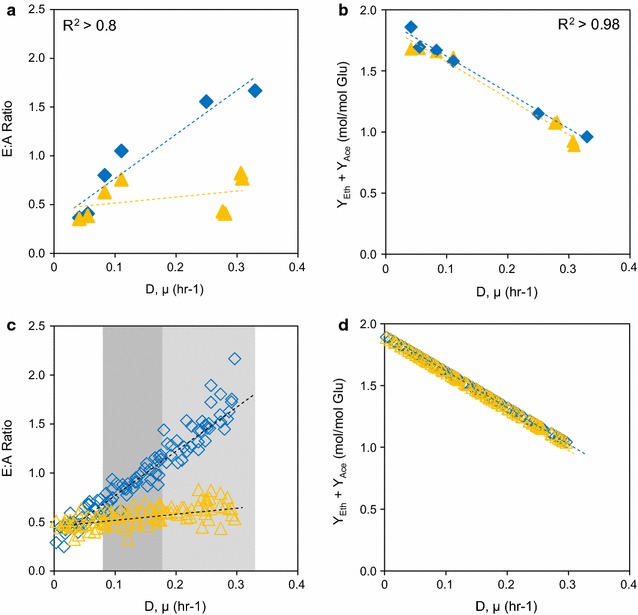
**a** Experimental data compilation of E:A ratios for cellulose (*triangles*) or cellobiose (*diamonds*). **b** Experimental data compilation of sum of ethanol and acetate yields from literature. **c** In silico implementation of E:A ratios. The *shaded regions* outline the points within the low growth, medium growth, and high growth sets. Symbols: cellulose (*triangles*) or cellobiose (*diamonds*). **d** In silico implementation of the sum of ethanol and acetate yields

Extensive compilation of fermentation data for cell growth in comparable conditions—equivalent defined media recipes with non-limited amounts of cellobiose or cellulose in both batch and continuous cultures under different growth (or dilution) rates—revealed several unique and interesting phenotypes regarding bioenergetics of *C. thermocellum* [9, 35, 36, 38, 44]. For instance, the ethanol to acetate (E:A) ratio is a commonly used indicator of bioenergetic balance in a given metabolic state of an anaerobic cell culture, where ethanol production is primarily tied to redox balance and acetate production is coupled with ATP synthesis. The experimentally observed E:A ratio differs substantially when wild-type *C. thermocellum* grew on various cellulosic substrates under various growth rates (Fig. 5a). Specifically, *C. thermocellum* could reach an E:A ratio upwards of two for growth on cellobiose, while the ratio never crested one for growth on cellulose. Interestingly, while the E:A ratios highly depend on the type of cellulosic substrate used, the sum of ethanol and acetate yields was inversely correlated with the growth rates, independent of which substrate was used (Fig. 5b). This also means that the correlation can serve as an ideal global constraint on bioenergetics of *C. thermocellum* and was employed for simulation in this study.

To better understand the bioenergetics of *C. thermocellum* when growing on different substrates under various growth rates, we sampled flux distributions based on experimental constraints followed by detailed analysis of the key cellular processes resulting in the observed trends of E:A ratios and sum of ethanol and acetate yields. Sampling is a common technique for examining a network structure to compare differences in conditions [57, 58] and/or infer regulatory elements [59].

We set a tight constraint on the sum of ethanol and acetate yields with respect to growth rates (Fig. 5d). We also introduced a noise level of 20 % to the E:A ratio at a given growth rate to account for variability among the E:A ratio parameters (Fig. 5c). The sum of yields and E:A ratios is considered jointly as the observed constraints below. For all sampling runs, the glucose equivalent uptake rates were randomly varied between the experimentally observed range of 5.0–7.5 mmol glucose equivalents/g DCW/h, but set equal for both cellobiose and cellulose cultures. This setup allowed us to obtain 100,000 individual yet comparable flux distributions across a range of growth rates that sufficiently covered the observed variance for both cellobiose and cellulose simulations. The distributions in fermentation products were distinct for each carbon source at different growth rates (Supplementary Figure S3 of Additional file 2). Increasing the number of sampling points to 500,000 did not have a significant effect on the flux trends, and so we are confident that these distributions are representative of cellular metabolism. The sampled flux distributions were analyzed to understand the metabolic differences which lead to the observed phenotypic differences between carbon sources.

#### Global redox and energy cofactor turnover

**Fig. 6:**
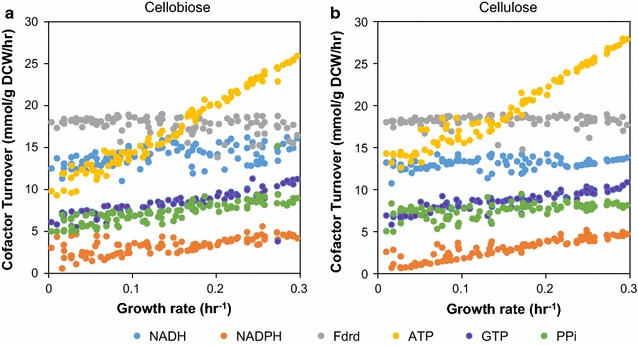
Mean cofactor turnover rates at various growth rates for *C. thermocellum* on cellobiose (**a**) and cellulose (**b**)

From the sampled flux distributions with the observed constraints, we first analyzed the turnover rates of the key metabolites ATP, GTP, pyrophosphate (PP_i_), reduced ferredoxin (Fd_rd_), NADH, and NADPH. It should be noted that a turnover rate of a metabolite determines how frequent that metabolite is biologically transformed and recycled at a given steady state and does not inherently give insight into the metabolite concentration within the cell [60]. The result shows that the turnover rates of ATP, GTP, PP_i_, and NADPH increased steadily with increasing growth rates (Fig. 6) as expected because the synthesis of biomass required these cofactors. The ATP turnover rate increased more sharply than GTP, PP_i_, or NADPH for both cultures, but slightly more for cellulose cultures which could be attributed to the additional burden of cellulosome synthesis and requirement of acetate biosynthesis. The ATP trend matched well with the experimental evidence reported by Zhang and Lynd [38].

We further analyzed the turnover rates of NADH and Fd_rd_ to illuminate the experimentally observed phenotypes. For growth on cellobiose, NADH turnover rates slightly increased as specific growth rates increased (Fig. 6), which correlated with the increased ethanol fluxes leading to higher E:A ratios. In contrast, the decrease in Fd_rd_ turnover rates manifested with a general decrease in hydrogen production, providing more electrons for ethanol biosynthesis (Additional file 2: Figure S3C). For growth on cellulose, the NADH and Fd_rd_ turnover rates were fairly constant across growth rates. These results suggest that the phenotypic constraints from growth on cellobiose lead to a restructuring of *C. thermocellum* redox metabolism, in particular NADH and Fd_rd_ turnover.

#### Central carbon metabolism

**Fig. 7:**
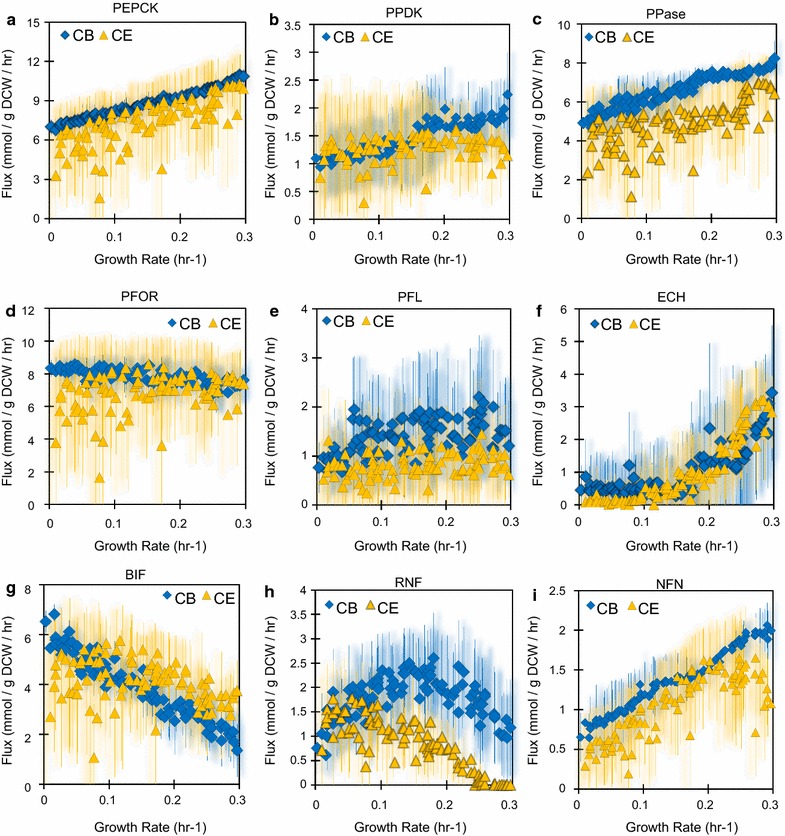
Sampled fluxes associated with bioenergetics of *C. thermocellum* for growth on cellobiose (CB, *blue*) and cellulose (CE, *orange*). The *icons* represent the mean flux for each sampled growth rate, while the *bars* represent the standard deviation across the samples. **a** PEPCK, PEP-carboxykinase. **b** PPDK, pyruvate:phosphate dikinase. **c** PPase, proton translocating pyrophosphatase. **d** PFL, pyruvate:formate lyase. **e** PFOR, pyruvate:ferredoxin oxidoreductase. **f** ECH, [NiFe] hydrogenase. **g** BIF, bifurcating hydrogenase. **h** RNF, reduced ferredoxin:NADH oxidoreductase. **i** NFN, NADH-dependent NADP^+^:ferredoxin oxidoreductase

We next examined the effect of the observed constraints on several key reactions of central carbon metabolism from phosphoenolpyruvate (PEP) to pyruvate to acetyl-CoA. For the conversion of PEP to pyruvate, simulations of both carbon sources predicted substantial flux through phosphoenolpyruvate carboxykinase (PEPCK), which is the first step in the malate shunt. The PEPCK activity increased with the increasing growth rates for both carbon sources even though the cellobiose simulations had a much tighter distribution (Fig. 7a). Direct conversion to pyruvate through pyruvate:pyrophosphate dikinase (PPDK), however, remained fairly constant for cellulose simulations but increased for cellobiose simulations with increasing growth rates (Fig. 7b). Regardless of cellulosic substrates and growth rates, PPDK fluxes were much lower than PEPCK fluxes. This simulation result clearly highlights the significant role of the PEPCK-dependent malate shunt on bioenergetics of *C. thermocellum* by generating energy in terms of GTP and producing NADPH from NADH, both of which are required for biomass synthesis and affect the experimentally observed ethanol production.

For the conversion of pyruvate to acetyl-CoA, the simulations for both carbon sources predicted fluxes through pyruvate:ferredoxin oxidoreductase (PFOR) were relatively constant under different growth rates, although the distribution was much wider for cellulose simulations (Fig. 7d). This might hint at less metabolic flexibility in the PFOR reaction when growing on cellobiose. Fluxes through pyruvate:formate lyase (PFL) were lower than PFOR fluxes in both conditions across growth rates (Fig. 7e), but the cellobiose cultures were predicted to have higher PFL fluxes than cellulose cultures. The latter implies that ethanol production might be limiting in cellobiose cultures because PFL is known to function as a metabolic valve to relieve redox imbalance [36].

#### Redox metabolism

We further examined the effect of the observed constraints on individual redox reactions. Regardless of cellulosic substrates, hydrogen production through the [NiFe] energy-conserving hydrogenase (ECH) increased almost exponentially as the growth rate increased, especially for E:A > 1 (or μ ≥ 0.18 h^−1^) (Fig. 7F). In contrast, hydrogen production through the bifurcating hydrogenase (BIF) dropped significantly for cellobiose with an increase in growth rates, yet it remained fairly consistent across growth rates for cellulose (Fig. 7g). This translates to a decrease in hydrogen production in cellobiose cultures while hydrogen production remains fairly constant in cellulose cultures (Additional file 2: Figure S3). The conversion of reduced ferredoxin to NADH through reduced ferredoxin:NADH oxidoreductase (RNF) was significantly greater for cellobiose simulations (Fig. 7h), particularly at high growth rates where cellulose simulations did not use RNF at all. Interestingly, the flux through RNF was parabolic in shape on cellobiose with increasing growth rate with an inflection point occurring with E:A ~ 1. Additionally, the flux through NADH-dependent reduced ferredoxin:NADP^+^ oxidoreductase (NFN) steadily increased with growth rate, because NADPH was required for anabolism (Fig. 7i) [61].

The observed constraints were hypothesized to increase the requirement for NADH in cellobiose cultures and the simulations corroborate this expectation, in particular by increasing the RNF flux and decreasing the BIF flux on cellobiose. Taken altogether, these results illustrate how *C. thermocellum* restructures its metabolism during growth on different carbon sources.

## Discussion

In this work, we have constructed the novel genome-scale model (GEM) of *C. thermocellum* DSM1313 *i*AT601. After extensive refinement with literature reports, we calculated the ATP requirements for growth-associated maintenance and cellulosome synthesis by fitting experimental data. With this model, we explored complex cellular phenotypes and model-guided strain design strategies for producing valuable chemicals. It is important to consider cellular phenotypes under different conditions to broadly understand and predict cellular behavior.

In particular, the cascade of carbon from PEP to pyruvate to acetyl-CoA in *C. thermocellum* provides key precursors for cell synthesis, and consists of alternative means of generating energy and shuttling electrons, especially when coupled to the complex redox metabolism [36]. Examining these reactions at various growth rates and on different substrates is an effective way to explore bioenergetics. Generally, the flux distributions from cellobiose simulations were less variable than flux distributions from cellulose simulations. This tightening of flux distributions implies that (i) bioenergetic constraints on carbon and electron flow limit the metabolic flexibility during growth on cellobiose, and/or (ii) that tighter regulatory mechanisms are imposed during growth on cellobiose than on cellulose at these key metabolic nodes.

### Proposed bioenergetic regulatory mechanism of *C. thermocellum* fermentation

Taken altogether, we can use the simulation results presented along with literature reports to propose a mechanism which explains the metabolic differences between cellobiose and cellulose cultures of *C. thermocellum*. Four key, interrelated motifs can help to shed light on this mechanism: Motif 1—energy modulation via acetate production, Motif 2—redox metabolism, Motif 3—regulation of PEP to pyruvate conversion, and Motif 4—PFL-dependent redox relief valve.

#### Motif 1

**Fig. 8:**
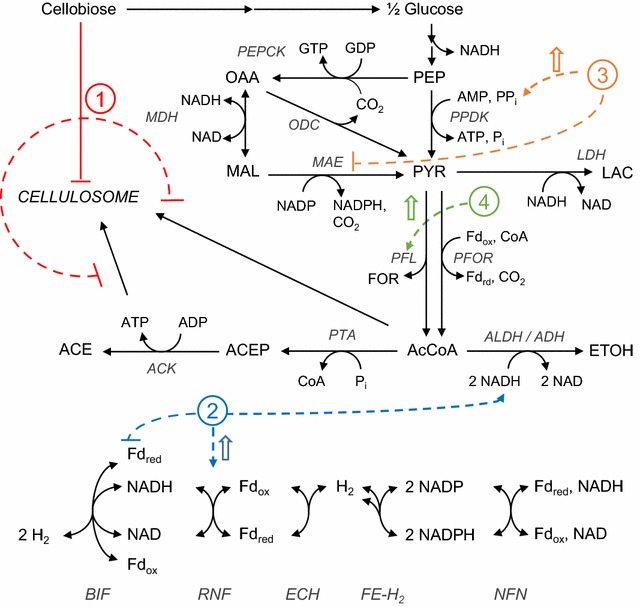
Proposed mechanism of bioenergetics influencing *C. thermocellum* during growth on cellulosic substrates. Motif 1: cellobiose inhibits cellulosome production, and the lower ATP requirement reduces the need for PTA-ACK. Motif 2: redox metabolism is restructured such that RNF activity is upregulated and/or BIF activity is downregulated to convert more reduced ferredoxin to NADH. Motif 3: the regulation of PEP to pyruvate is affected by PP*i* concentrations, and increased RNF activity can be used to synthesize PP_i_. Motif 4: PFL acts as a redox relief valve and is likely activated by a redox imbalance

Energy modulation via acetate production is one of the critical motifs regulating bioenergetics of *C. thermocellum*. Acetate production during growth on cellobiose likely drops because less PTA-ACK activity would be necessary to generate the required ATP for cellulosome synthesis. Indeed, it has been estimated previously that the amount of ATP required for cellulosome synthesis and the amount of ATP produced by PTA-ACK are roughly equivalent [38]. While not necessarily indicating causation, this underlying assumption is highlighted by the cofactor turnover in our simulations, where ATP turnover is higher for cellulose than cellobiose simulations (Fig. 6). It has been experimentally observed that cellobiose inhibits synthesis of the cellulosome at both enzymatic [35] and transcriptomic levels [62] (Fig. 8); however, real-time PCR has shown little difference in PTA-ACK expression between cellulose and cellobiose cultures [63] which suggests that cellobiose is not a direct regulator of acetate synthesis. Alternatively, elimination of hydrogen production leads to diminished acetate production in *C. thermocellum*, and it has been proposed that electron perturbations are more influential than PTA-ACK perturbations on ethanol production [36].

#### Motif 2

Redox metabolism of *C. thermocellum* is very robust and a critical motif in controlling cellular bioenergetics. To account for high E:A ratios for growth on cellobiose, the cell must have an ample supply of NADH. This is facilitated by an increase in RNF and decrease in BIF activities on cellobiose as observed in the simulations (Figs. 7g, h, 8). An increase in RNF flux will also limit the NADH available for hydrogen synthesis by BIF. RNF is expressed during batch growth on cellulose [64], and in chemostats expression is significantly higher for cellobiose cultures than for cellulose cultures across growth rates [62]. Thus, it is feasible that cellobiose can activate RNF expression, in an opposite phenotype to cellulosome synthesis. Further, the low flux through RNF in all simulations suggests that RNF may be limiting ethanol production by throttling NADH generation.

#### Motif 3

The motif of PEP to pyruvate conversion in *C. thermocellum* enables it to efficiently regulate cellular bioenergetics and carbon flux. Our simulations suggest a link between RNF and PP_i_ by ways of the conversion of PEP to pyruvate. The RNF protein complex is imbedded in the membrane and couples proton export to ferredoxin oxidation to form NADH. The generated proton motive force can be used to drive ATP and/or PP_i_ synthesis [29, 64], which is consistent with the higher PPase flux in cellobiose culture simulations (Fig. 7c). Experimentally, it has been shown with in vitro purified enzyme assays that a high concentration of PP_i_ can enhance PPDK activity and inhibit MAE activity [65]. It is interesting that the allosteric control of PP_i_ on PPDK and MAE was not included in the model, yet the constraints on ethanol and acetate manifest into an observed increase in PPDK activity in cellobiose simulations (Fig. 8). An additional consequence of more PPDK flux means less NADH is converted to NADPH through the malate shunt, and the additional NADH can then go towards ethanol production.

#### Motif 4

PFL has been described as a redox relief valve in *C. thermocellum* [36]. For context, as described above, RNF produces NADH and oxidizes ferredoxin. It has been suggested previously that RNF is the major bottleneck in ethanol production, and the limiting capacity of RNF causes an accumulation of reduced ferredoxin which then leads to an increase in PFL flux [36]. This idea comes from experimental evidence where suppressing hydrogen production via chemical inhibitors or genetic manipulations leads to an increase in formate production on cellobiose, and the PFL reaction has been described as an overflow reaction used to generate acetyl-CoA from pyruvate without generating reduced cofactors [32, 66]. Interestingly, PFL and its activating enzyme are highly expressed across multiple conditions [30, 64, 67], even when no formate production is observed. Expression of PFL without formate production implies a redox related, possibly allosteric, regulatory mechanism (Fig. 8). While formate production was not reported in most of the training data, in our simulations the cellobiose set has a consistently higher PFL flux (Fig. 7e). The importance of PFL in the production of acetyl-CoA from pyruvate can also be seen when eliminating PFL activity through chemical inhibition or genetic manipulation, which is shown to increase lactate production more than ethanol production [18, 32]. Under conditions of redox stress, it would be more beneficial to produce ethanol and consume 2 NADH than to produce lactate and consume 1, particularly if PFL is used to generate acetyl-CoA without producing reduced cofactors. However, if PFL activity is not possible, *C. thermocellum* cannot completely balance carbon and redox cofactors to produce ethanol and cell growth, which stalls the conversion of pyruvate to acetyl-CoA and leads to lactate production.

### Relation between ethanol production and cellulose degradation

Similar to PFL, alcohol dehydrogenases (ADHs) responsible for ethanol production are also seen to be highly expressed under multiple conditions, although ethanol production varies [30, 64]. The availability of reduced ferredoxin or NADH could then feasibly activate ethanol and formate production as an overflow to relieve redox stress. The availability of NADH as the main activator of ethanol synthesis also makes sense when considering that addition of methyl viologen to cellobiose chemostats led to an increase in ethanol production without significant increase in transcription of ethanol synthesis genes [68], since methyl viologen would oxidize ferredoxin and the NADH produced in glycolysis would not be oxidized along with reduced ferredoxin via the bifurcating hydrogenase. More evidence to the overflow behavior of ethanol and formate production can be seen when growing cells in continuous cultures with a lower cellodextrin feed concentration. Under low substrate conditions in rich media, ethanol and formate production are very low, and acetate is the major fermentation product, regardless of using cellobiose or cellulose as a carbon source [63].

The simulation results and literature summaries presented above offer some interesting suggestions. To properly understand why cellobiose cultures produce so much more ethanol, it is useful to think of *C. thermocellum* in its native environment, i.e., degrading complex biomass in a co-culture in soil [69, 70], where there is not likely to be a substantial cellobiose concentration. In fact, in designed co-cultures, *C. thermocellum* prefers to make acetate and hydrogen if these products can be consumed by its cohabitant [71]. Isolated growth on cellobiose, however, can be considered a perturbation away from the native environment since high concentrations of cellobiose have been shown to repress cellulosome synthesis [35]. This repression is key to the increased ethanol production on cellobiose, because by lowering the cellulosome burden by tenfold (i.e., from 20 % DCW to 2 % DCW), our calculations above estimate that the cell needs to produce about 11 mmol ATP/g DCW/h less on cellobiose.

Without the ATP burden of cellulosome synthesis, less flux through PTA-ACK is needed, and this triggers a dramatic restructuring of carbon and electron fluxes to maintain the rates of glycolysis examined here. As a result, there is an increase in RNF flux, which leads to more PPDK flux. Both these reactions can enhance the supply of NADH and lead to the observed overflow of ethanol production on cellobiose. As RNF reaches its maximum capacity, PFL flux increases to balance the need for acetyl-CoA and the redox state of the cell.

Generally, these results indicate that for growth on cellulose, a high glycolytic flux and sufficient conversion of reduced ferredoxin to NADH (e.g., by eliminating hydrogen production or overexpressing RNF) will be critical for high ethanol production. A significant level of control is necessary to accomplish this goal, although it is still unclear at this point how exactly the proposed motifs are controlled with respect to each other, or what additional regulatory elements might be active with, or instead of, the proposed mechanism above. There are still many questions regarding the bioenergetic control mechanisms which balance carbon and electron fluxes in *C. thermocellum*. However, we believe these questions can be addressed as more OMICs and fermentation datasets become available and are integrated.

## Conclusions

Our genome-scale model for *C. thermocellum*, with its dynamic cellulosome component, displays a significant increase in function and predictive capability to the previous model. We built and extensively refined this model using the KEGG database and our previously reported core model [36]. From this draft, we were able to tune the ATP cost for growth-associated maintenance and cellulosome synthesis and secretion. Using these tuned parameters, we further validated the model by quantifying the difference in bioenergetics of cellodextrin utilization in silico. These results matched experimental data well [38]. As an additional assessment, we provided a view into the potential of this model for strain design applications by calculating cMCS strategies for the production of ethanol, hydrogen, and isobutanol.

We then used the model to address a few fundamental questions about *C. thermocellum* metabolism which arose after compiling multiple sets of training data. Namely, what is the mechanism behind the increase in ethanol production when growing on cellobiose versus cellulose? Using our GEM, we sampled 100,000 flux distributions for simulations on cellobiose and cellulose which were constrained to experimental data. By examining the difference between the carbon sources at different growth rates, we show how ethanol production in *C. thermocellum* is part of the overflow metabolism when growing on cellobiose.

We envision that with the tuned GEM *i*AT601, *C. thermocellum* can be designed as a manufacturing CBP platform to produce a large space of potential biofuels and chemicals beyond ethanol, isobutanol, and hydrogen using the modular cell design concept [72]. Altogether, the GEM presented here will be a useful tool for further investigating cellular phenotypes and model-guided strain design.

## Methods

### Construction of draft metabolic model

A workflow of the model construction process is given in Fig. 1a. The first draft of the metabolic network of DSM 1313 was constructed using the automatic reconstruction function *getKEGGModelForOrganism()* of the RAVEN toolbox [73]. This function compiled reactions from the KEGG database organism entry for DSM1313 (T01933, *ctx*) with the complete set of coding sequences from the genome assembly (GenBank: CP002416.1) so that each reaction is linked to a specific protein encoded by the genome [33]. This draft reconstruction contained gene--protein relationships (GPR), pathway information, and reaction stoichiometry.

Transport reactions were linked to genes by compiling a list of putative transporters and then curating reactions via manual inspection. The list was compiled by three methods. The first method used InterProScan 5 [74, 75] to find 169 putative transporters within the DSM 1313 protein sequences. The second method extracted annotated transporters from alternative *Clostridial* GEMs [26, 76, 77] and subjected them to reciprocal blast hit (RBH) and *hmmscan* [78] to determine similar genes in DSM 1313. For RBH, we used 1e-50 and blast length of fifty amino acids as cut-offs. The third method used the Transporter Substrate Database [79] to extract protein sequences for each substrate exchange reaction within the model and compared them to the genome sequence of DSM 1313 with a cutoff of one for *hmmscan* and 1e−50 for RBH.

Further, as it is known that *C. thermocellum* cofactor specificity in glycolysis is atypical [29], we investigated reaction sets from the automatic reconstruction which only differed by cofactor choice, e.g., NADH versus NADPH or ATP versus GTP. Enzymes with available in vitro data were adjusted accordingly [29]. For enzymes which the automatic reconstruction predicted equivalent reactions with different cofactors, the proteins linked to these reactions were analyzed for cofactor specificity using Cofactory [40]. This software determines the specificity towards NAD, NADP, or FAD of proteins by predicting Rossmann folds from primary sequence. Equivalent reactions with different cofactors were consolidated in the model according to Cofactory results.

### Refinement of genome-scale metabolic model

The KEGG draft network contained many gaps in the central metabolism and was plagued by unrealistic predictions because it assumed that many reactions were reversible, which lead to thermodynamically infeasible pathways. To build a working GEM, the KEGG draft network was expanded and refined in the following manner:

i. The central metabolic network recently reported [36] was manually built into the GEM, filling in gaps in glycolysis and redox metabolism which were not automatically included.
ii. We adapted the dry cell weight composition presented for strain ATCC 27405 [26] to reflect the differences in genomic content between strains as well as a stringent calculation of ATP requirements for biomass synthesis [45, 46], then included the cell composition reaction into the network (Table 4).

**Table 4:** Comparison of ATP requirements for *C. thermocellum* and model organisms during anaerobic growth

	ATP requirement (mmol ATP/g DCW)		
	*C. thermocellum*	*E. coli* [46]	*S. cerevisiae* [99]
Polysaccharides/cell wall	7.16	2.05	4.63
Protein			
Amino acid synthesis	5.65	1.36	1.81
Polymerization	17.49	19.14	17.76
Lipid	0.52	0.14	0
RNA			
Nucleoside monophosphate formation	1.20	3.45	1.35
Polymerization	0.27	0.92	
mRNA turnover	0.30	1.39	0.71
DNA			
Deoxynucleoside monophosphate formation	0.48	0.86	
Polymerization	0.18	0.19	
Subtotal	33.23	29.50	26.26
Transport			
Ammonium	8.43	4.24	6.29
Potassium	0.20	0.19	2.4
Phosphate	0.80	0.77	
Total ATP required	42.66	34.71	34.95
*Y*_ATP_^MAX^ (g DCW/mol ATP)	23.44	28.81	28.61
iii. We added in several artificial reactions to convert identical yet alternately described metabolites (e.g., beta-d-fructose 6-phosphate → d-fructose 6-phosphate) within the network to efficiently close gaps between discrepancies.
iv. We used the automatic gap filling function of the RAVEN Toolbox and the previously constructed *C. thermocellum* GEM *i*SR432 from Roberts et al. [26] as a template to increase network connectivity.
v. We added reactions to fill gaps in the sulfate utilization pathway, which is known to be utilized as a sole sulfur source [80], as well as shikimate kinase, homoserine kinase, and spontaneous glutamate semialdehyde cyclization reactions to allow synthesis of all essential amino acids in minimal media.
vi. We manually inspected each reaction in the network for appropriate reversibility, adjusting reactions outside of glycolysis and substrate-level phosphorylation to only consume ATP. This convention is commonplace [81] and it removed the cycles in the model which were incorrectly generating energy. During the manual curation process, extensive metadata were included for reactions and metabolites to allow for cross linking between KEGG [82], MetaCyc [83], MetaNetX [84, 85], SEED [86], BRENDA [87], and other databases.

The refined model is included in the Supplementary Materials and has been deposited in the DOE KBase (https://narrative.kbase.us/narrative/ws.13674.obj.2).

### Metabolic network analysis

Metabolic network analysis is a powerful tool for studying cellular phenotypes and model-guided strain design. In our study, both flux balance analysis (FBA) and elementary mode analysis (EMA)-based techniques were employed to analyze the GEM *i*AT601. In general, a metabolic network can be represented with a stoichiometric matrix $S \in {\mathbb{R}}^{m x n}$, consisting of *m* metabolites and *n* reactions, such that the entry *s*_*i,j*_ is the stoichiometric coefficient of metabolite *i* in reaction *j*. A valid flux distribution vector $v \in {\mathbb{R}}^{n x 1}$ satisfies a steady-state condition

1 $$\varvec{S } \cdot \varvec{v} = 0$$

and is thus constrained by mass balance. The flux distribution vector is also constrained by thermodynamics such that

2 $$v_{j} \ge 0$$

for all irreversible reactions *j*.

FBA is a commonly used computational tool using stoichiometric and thermodynamic constraints to optimize a cellular objective, such as maximum cell growth [88]. In our study, the COBRA [89] and RAVEN [73] toolboxes within the MATLAB environment (MathWorks, Natick, MA) were used to perform FBA-based computations. The algorithm parameters were set to remove any Type III (internal) loops [90] within the solution. Changes in media recipes were implemented as bounds on nitrogen and sulfur sources. For MTC media [44, 91], urea and ammonia were available as a nitrogen source, while sulfate and cysteine were available as a sulfur source. The cysteine uptake rate was bound at 0.5 mmol/g DCW/h while rates of all other species were unbound. For low-carbon (LC) media [44], cysteine was the sole sulfur source while ammonia was the sole nitrogen source. Cofactor turnover rates were calculated using Flux-Sum Analysis (FSA) [60]. For any FBA solution, the flux-sum $\varPhi_{i}$, or turnover rate (mmol/g DCW/h), of metabolite *i* can be calculated by

3 $$\varPhi_{i} = 0.5 \mathop \sum \limits_{j} \left| {s_{i,j} \cdot v_{j} } \right|$$

for all reactions *j* in which the metabolite participates.

EMA seeks to find all solutions to Eqs. 1 and 2 that are subject to an additional decomposability constraint [92]. The set of solutions is called elementary modes. Using the set of elementary modes, one can calculate the minimal number of reaction deletions needed to guarantee coupled product and cell yield [51]. These genetic modification target groups are called constrained minimal cut sets (cMCS) [50]. EMA and cMCS calculations have typically been computationally prohibitive for large networks; however, recent progress has been made in algorithm improvement for both methods. For the calculation of elementary modes of the GEM, we used the algorithms recently developed [93, 94]. To calculate cMCS, we required cell growth to be greater than 0.0001 and specified a minimum product yield of 60 % theoretical maximum using the recently developed cMCS method of von Kamp and Klamt [54]. All calculations were performed in Matlab on a desktop PC (3.4 GHz 4 core processor, 32 GB RAM). Additional file 3 contains a list of all calculated cMCS strategies for ethanol, hydrogen, and isobutanol.

### Calculation of experimental yields and fluxes for model constraints

Experimental data were obtained from multiple sources (see “Results”), and as such not all extracellular metabolites were measured under different conditions. However, for a given batch experiment, concentrations of various metabolites were determined at multiple time points during exponential growth. Fluxes were calculated as in our previous work [36] from these concentration profiles as follows:

4 $$\upsilon_{P} = \mu \cdot Y_{P/X} = \mu \cdot \frac{{{\text{dC}}_{P} /{\text{d}}t}}{{{\text{dC}}_{X} /{\text{d}}t}}$$

where υ_p_ (mmol/g DCW/h) is the specific rate (or flux) of metabolite *P*,* μ *(h^−1^) is the specific growth rate, *Y*_*P*/*X*_ is the yield of metabolite *P* per unit DCW *X*, and C_*P*_ (mmol/L) and C_*X*_ (g DCW/L) are concentrations of *P* and *X*, respectively.

For chemostat cultures, the fluxes were calculated as follows:

5 $$v_{P} = D \cdot Y_{P/X} = D \cdot \frac{{C_{P,out} - C_{P,in} }}{{C_{X,out} - C_{X,in} }}$$

where *D* is the dilution rate (h^−1^).

### Implementation of the cellulosome in the genome-scale model

The cellulosome is the cellulose-degrading protein complex covalently bound to the surface of certain cellulolytic bacteria, such as *C. thermocellum*. The previous GEM of *C. thermocellum*, *i*SR432 [26, 27], roughly included the cellulosome on top of the dry cell weight (DCW) reaction in a condition-independent manner. However, it is well documented that the cellulosome fraction of total DCW changes from 2 to 20 % when growing on cellobiose versus cellulose, respectively [35], and the protein composition of the cellulosome itself changes when growing on alternative substrates [43, 62, 67, 95, 96]. Therefore, our cellulosome reaction was set up to allow for dynamic switching between modeling cellobiose- versus cellulose-consuming growth conditions. This is an important distinction due to the increased ATP requirement for exporting more protein from the cell.

The fractional composition for dry cell weight was 0.5285 g protein + 0.026 g DNA + 0.0655 g RNA + 0.076 g lipid + 0.2242 g cell wall + 0.00494 g solute pool + 0.0304 g total_LTA → g dry cell weight (DCW). A second reaction was implemented to combine the DCW term and the cellulosome term, and this whole cell term was adjusted depending on carbon source, specifically: 1 g DCW + 0.02 g cellulosome term → biomass for cellobiose cultures and 1 g DCW + 0.2 g cellulosome term → biomass for cellulose cultures [26].

The composition of the cellulosome was initially set equivalent to the protein term. Using experimentally observed cellulosomal protein abundances, we altered the cellulosome composition systematically. First, a matrix ***A*** was created by counting the amino acids required to synthesize cellulosomal proteins. The entry *A*_*i,j*_ corresponds to the number of amino acid *i* encoded by the sequence of protein *j*. Second, the abundances of each protein were condensed into a condition-specific vector ***c*** normalized to CipA as presented by Raman et al. [43]. The total amino acid count across all cellulosomal proteins for any condition can be calculated by ***A*·*c***. Finally, the condition-specific amino acid count is converted to mmol/g cellulosome similar to the calculation of protein or DNA terms (Additional file 1).

To complement the adjustable cellulosome reaction, transport reactions were added for cellodextrin oligomers of length 3–6 glucose subunits, i.e., cellotriose (G3) to cellohexaose (G6). Glucose and cellobiose transport were included in the automatic reconstruction. It has been shown that *C. thermocellum* cellodextrin transporters prefer longer chain oligomers [42]. Further, there is a complex set of regulatory interactions where excess cellobiose represses cellulase activity [35] and, conversely, cellobiose uptake is inhibited by the presence of G3 to G5 oligomers [56]. Upon entering the cell, cellodextrins are cleaved in a phosphorolytic manner [37] and so reactions were included to utilize G6 to G2 oligomers by a sequential chain-shortening pathway generating glucose-1-phosphate and a cellodextrin of length G(N-1). The final glucose residue in this depolymerization pathway is phosphorylated with ATP. This mechanism of transport and phosphorolytic cleavage costs two ATP per cellodextrin imported, regardless of length, and as such the ATP yield per glucose equivalent is higher when assimilating longer oligomers [38].

It is difficult to obtain information regarding individual oligomer uptake rates in vivo, so most studies report data in the units of mmol glucose equivalents/g DCW/h. To utilize this information as a constraint in the GEM, we implemented a flux ratio constraint [97] between the individual cellodextrin uptake reactions and the uptake rate of glucose equivalents as such

6 $$6{\mathbf{r}}_{\text{G6}} + { 5}{\mathbf{r}}_{\text{G5}} + { 4}{\mathbf{r}}_{\text{G4}} + { 3}{\mathbf{r}}_{\text{G3}} + { 2}{\mathbf{r}}_{\text{G2}} + { 1}{\mathbf{r}}_{\text{G1}} = {\mathbf{r}}_{\text{Glu Eq}}$$

where **r**_G(*N*)_ is the specific uptake rate of the cellodextrin of length *N*. This constraint maintains stoichiometric balance when using commonly reported experimental data to test the model.

Finer details into the model structure are included in Additional file 1.

### Calculation of ATP cost

To tune the ATP growth-associated maintenance requirement, we performed a series of optimizations to fit experimental data from cellobiose-grown batch cultures. Initially, non-growth-associated maintenance (NGAM) was set at 3.27 mmol ATP/g DCW/h [38] and no growth-associated maintenance (GAM) was specified. To fit experimental data, the GAM was varied between 1 and 50 mmol ATP/g DCW/h [76], and the cellulosome ATP requirement for synthesis was identical to the protein term of the biomass reaction (43.28 mmol ATP/g Protein/h, Additional file 1).

To tune the ATP cost of cellulosome synthesis, the coefficient for ATP in the cellulosome synthesis reaction was varied from 40 to 100 mmol ATP/g cellulosome/h while optimizing for maximal growth and maintaining experimental constraints, similar to above. Tuning these additional ATP requirements allowed finding the best fit to experimentally observed growth rate. All subsequent simulations used these parameters.

### Flux sampling based on ethanol and acetate production

Investigation of the difference in ethanol production between culture conditions was performed by constraining ethanol and acetate production as a function of cell growth. To implement these constraints, we used a series of flux ratios. First, to constrain the sum of ethanol and acetate yields, we calculated the following flux ratio:

7 $$Y_{E/G} + \, Y_{A/G} = \, - 2.9 \, \mu \, + \, 1.9$$

8 $${\text{r}}_{\text{E}} + {\text{ r}}_{\text{A}} = \, \left( { - 2.9 \, \mu \, + \, 1.9} \right) \, \times {\text{ r}}_{\text{G}}$$

where the slope and intercept were obtained from the linear relationship in Fig. 5b Second, to constrain the ethanol to acetate (E:A) ratio, we similarly calculated the following flux ratio:

9 $${\text{r}}_{\text{E}} /{\text{ r}}_{\text{A}} = m \times \, \mu \, + b$$

where the slopes and intercepts for cellobiose and cellulose were obtained from the relationships in Fig. 5a. Since the experimental data displayed some variance in this parameter, we wanted to ensure a complete representation of cell phenotypes. To accomplish this, we introduced approximately 20 % noise into our constraint by randomly varying the slope and intercept.

Given the variability in reported E:A ratios, we chose to sample the phenotypic space of cellobiose and cellulose cultures given the constraints above to minimize bias between flux distributions. To perform the sampling, we first randomly generated 100 normally distributed values of μ between 0 and 0.3 (h^−1^) as well as 100 values for the glucose uptake rate between 5.5 and 7.5 mmol glucose equivalent/g DCW/h, the range seen across multiple datasets. For each growth rate and glucose uptake rate, the sum of ethanol and acetate yields and E:A ratios was calculated. All other fermentation products were unconstrained. The uniform sampling was performed using *optGpSampler* [98] with a step size of 1000 for each growth rate. Retaining 1000 flux distributions at each growth rate gave a set of 100,000 flux distributions for both cellobiose and cellulose simulations. Multiple sets of sampling simulations with differing numbers of retained distributions did not affect the reaction trends presented (data not shown).

## Additional files

10.1186/s13068-016-0607-x Model structure and biomass term calculations.
10.1186/s13068-016-0607-x Supplementary figures and descriptions.
10.1186/s13068-016-0607-x Minimal cut sets for ethanol, hydrogen, and isobutanol production.
10.1186/s13068-016-0607-x The model in SBML format set up for growth on cellobiose.
10.1186/s13068-016-0607-x The model in SBML format set up for growth on cellulose.

## Abbreviations

CBP: consolidated bioprocessing
FBA: flux balance analysis
GEM: genome-scale metabolic model
NGAM: non-growth-associated maintenance
GAM: growth-associated maintenance
DCW: dry cell weight
EMA: elementary mode analysis
cMCS: constrained minimal cut set
G1: glucose
G2: cellobiose
G3: cellotriose
G4: cellotetraose
G5: cellopentaose
G6: cellohexaose
EA: ethanol to acetate
PPi: pyrophosphate
Fd_rd_: reduced ferredoxin
PEP: phosphoenolpyruvate
PEPCK: phosphoenolpyruvate carboxykinase
PPDK: pyruvate:pyrophosphate dikinase
MDH: malate dehydrogenase
MAE: malic enzyme
PFOR: pyruvate:ferredoxin oxidoreductase
PFL: pyruvate:formate lyase
ECH: [NiFe] energy-conserving hydrogenase
RNF: ferredoxin:NADH oxidoreductase
NFN: NADH-dependent ferredoxin:NADP^+^ oxidoreductase
