# Supplementary material for: Exploring complex cellular phenotypes and model-guided strain design with a novel genome-scale metabolic model of Clostridium thermocellum DSM 1313 implementing an adjustable cellulosome
Source: Biotechnol Biofuels. 2016 Sep 6;9(1):194. doi: 10.1186/s13068-016-0607-x (PMC5012057; doi:10.1186/s13068-016-0607-x)

A Novel Genome Scale Metabolic Model of *Clostridium thermocellum* DSM 1313 Implementing an Adjustable Cellulosome for Examining Complex Cellular Phenotypes and Model-Guided Strain Design

Supplementary File S2

R. Adam Thompson[1,2], Sanjeev Dahal[2,3], Sergio Garcia[2,4], Intawat Nookaew[2,3], and Cong T. Trinh[1,2,4*]

[1]Bredesen Center for Interdisciplinary Research and Graduate Education, University of Tennessee, Knoxville, TN 37996, USA and Oak Ridge National Laboratory, Oak Ridge, TN 37831, USA

[2] BioEnergy Science Center, Oak Ridge National Laboratory, Oak Ridge, TN 37831, USA

[3]Comparative Genomics Group, Biosciences Division, Oak Ridge National Laboratory, Oak Ridge, TN, 37831

[4]Department of Chemical and Biomolecular Engineering, University of Tennessee, Knoxville, TN 37996, USA.

*Corresponding author: ctrinh@utk.edu

Supplementary Table S1. Comparison of *i*AT601 predictions of mutant strain growth using the calculated GAM parameter. Fermentation product fluxes were constrained using experimental values presented in Thompson et al, *Met. Eng.*, 2015, **32**:207-219. Gene deletions were implemented by constraining appropriate reaction bounds to zero.

	**Specific growth rate μ (hr^{-1})**	
**Strain**	**Experiment**	**Simulation**
Parent	0.33	0.33
Δ*hydG*	0.24	0.26
Δ*hydG* Δ*ech*	0.22	0.25
Δ*hydG* Δ*pta-ack*	0.16	0.18

Supplementary Figure S1. Distribution of amino acids per unit cellulosome for growth of *C. thermocellum* on various cellulosic substrates. Abbreviations: Cb, cellobiose; C, crystalline cellulose (Avicel); CX, cellulose + xylan; CP, cellulose + pectin; CPX, cellulose + pectin + xylan; SWG, pretreated switchgrass; ZT, amorphous cellulose (Z-Trim®); and Cell, median values across all other amino acid distributions and the values used in our cellulosome term. Data adapted from Raman et al, *PLoS ONE*, 2009, **4**:e5271.

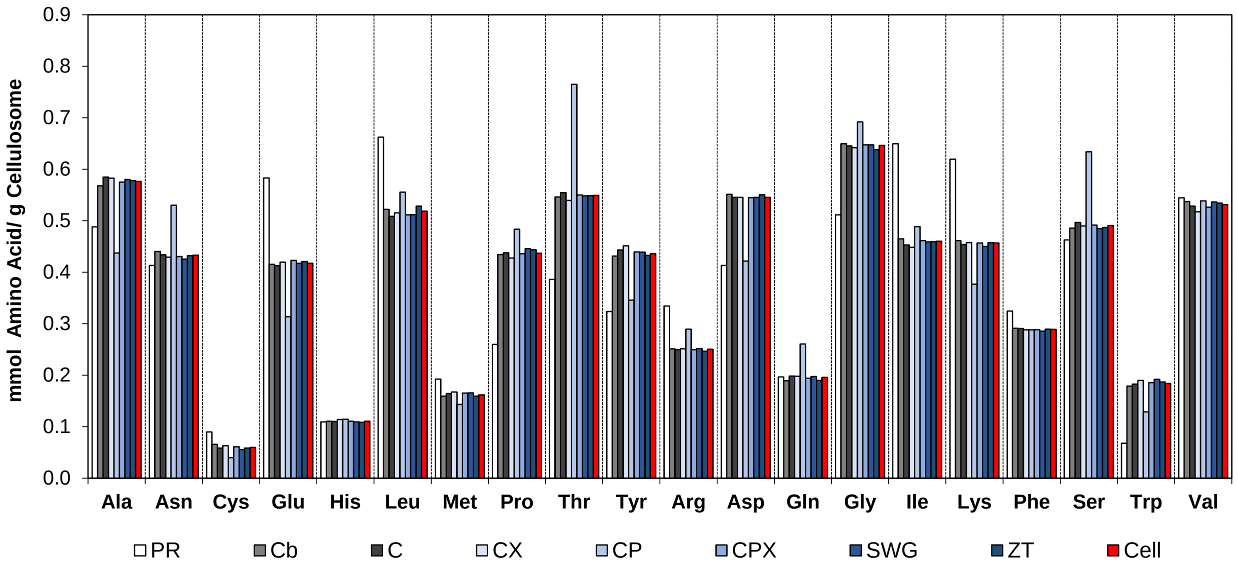

Supplementary Figure S2. Fractional reaction appearance in ethanol constrained Minimal Cut Set strategies for reactions with a fraction above 1%. Fractional appearance calculated by summing the times a reaction is present in an MCS divided by the total cMCS count.

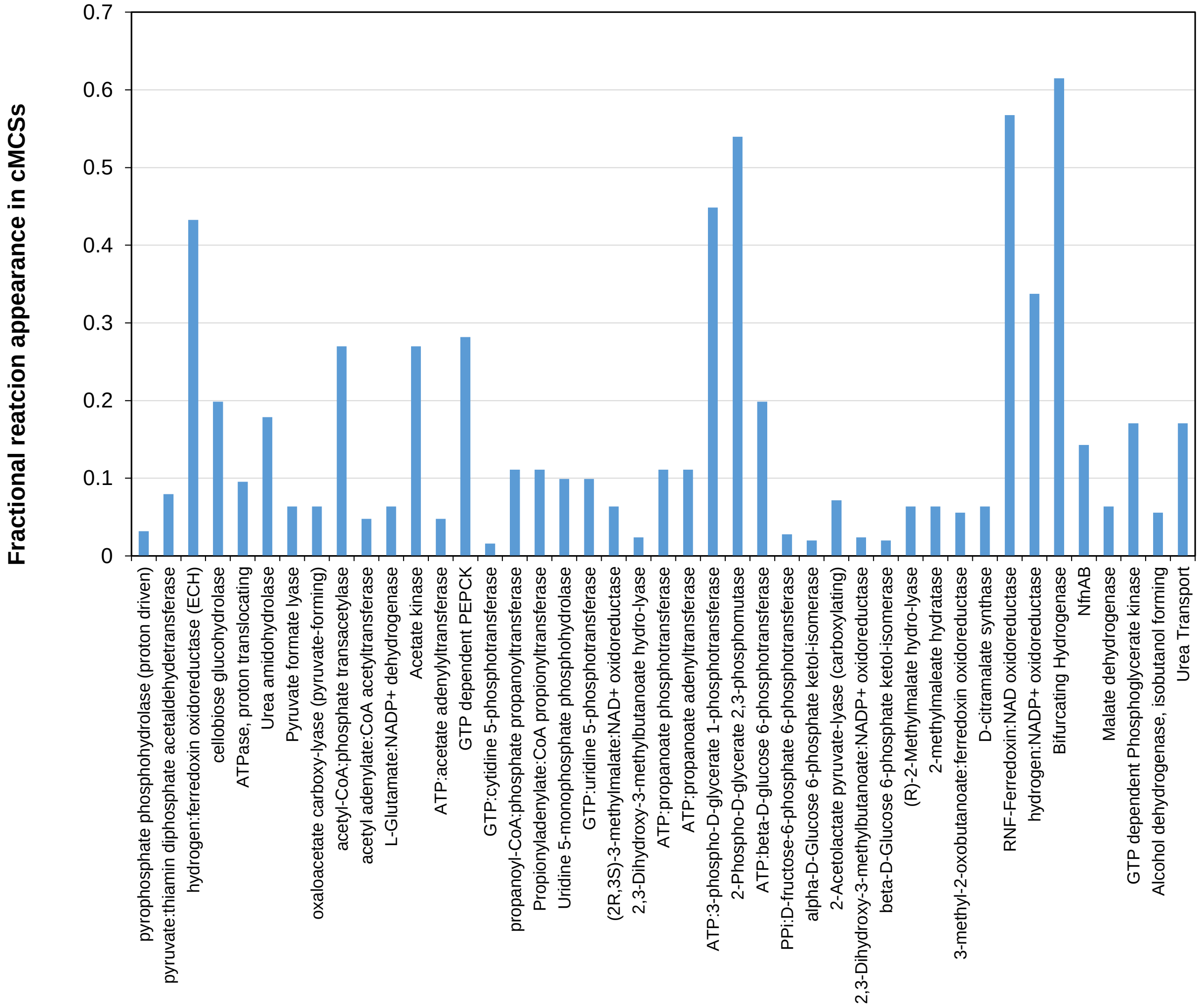

Supplementary Figure S3. Predicted averaged fluxes of major fermentation products for growth of *C. thermocellum* on cellobiose (**A-C**) and cellulose (**D-F**). These fluxes were presented for low growth phase (**A, D**), moderate growth phase (**B, E**), and high growth phase (**C, F**).

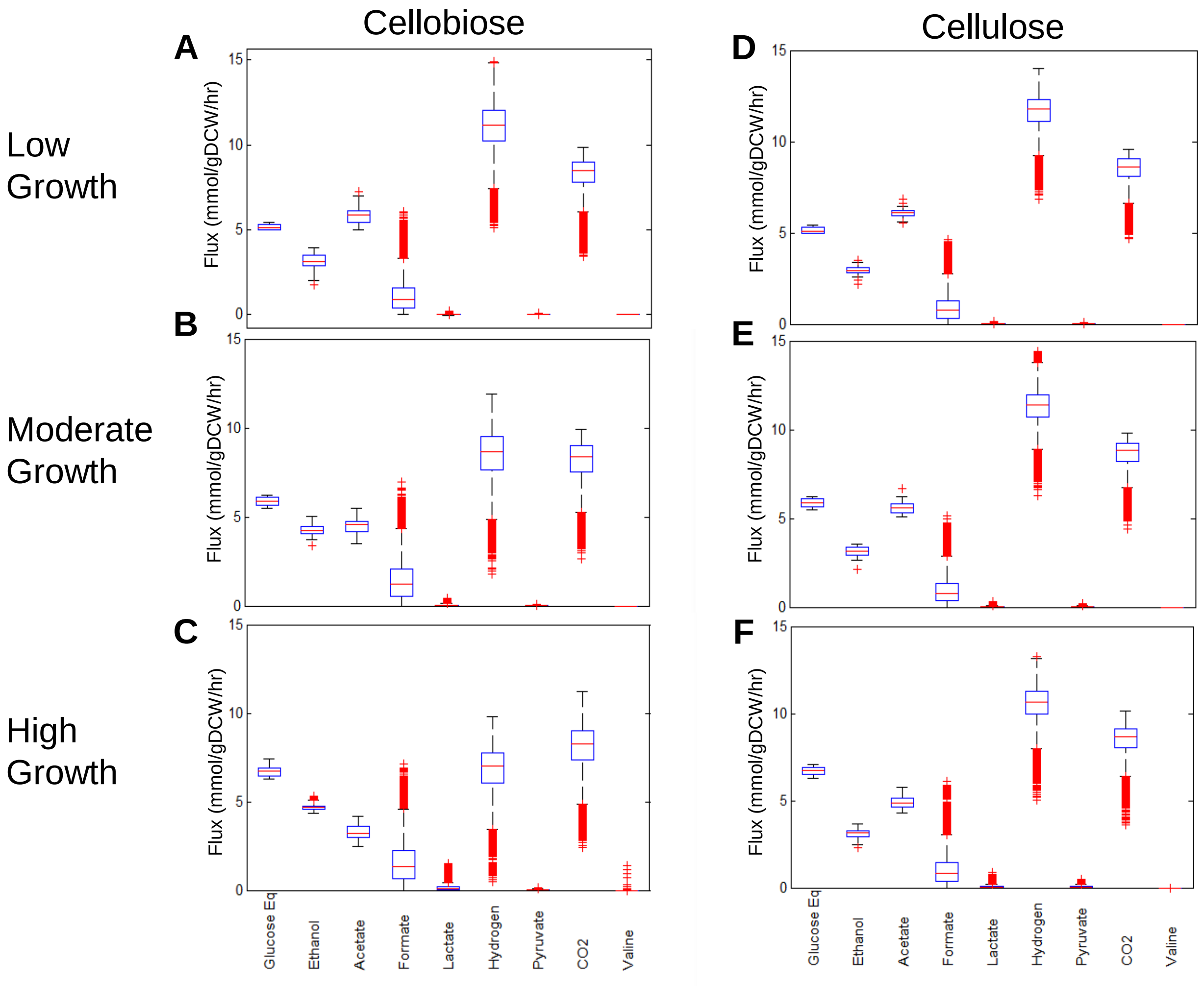

Supplement: Supplementary file 2 — 10.1186/s13068-016-0607-x Supplementary figures and descriptions. [file 13068_2016_607_MOESM2_ESM.pdf]
